# Acute Coronary Syndromes: State-of-the-Art Diagnosis, Management, and Secondary Prevention

**DOI:** 10.3390/jcm15010016

**Published:** 2025-12-19

**Authors:** Xun Yuan, Stephan Nienaber, Ibrahim Akin, Tito Kabir, Christoph A. Nienaber

**Affiliations:** 1 Department of Cardiology, Royal Brompton and Harefield Hospitals, Guy’s and St Thomas’ NHS Trust Foundation, London SW3 6NP, UK; xun.yuan@nhs.net (X.Y.);; 2 Department of Cardiology, Milton Keynes University Hospital, Milton Keynes MK6 5LD, UK; 3 Department of Cardiology, Heart Centre Cologne, Faculty of Medicine and University Hospital, University of Cologne, 50937 Cologne, Germany; 4 Department of Cardiology, University Hospital Heidelberg-Campus Mannheim, 69120 Mannheim, Germany

**Keywords:** acute coronary syndrome, myocardial infarction, dual antiplatelet therapy, PCI, cardiogenic shock, dyslipidaemia, precision medicine

## Abstract

**Background:** Acute coronary syndromes (ACSs) remain a leading cause of death and disability. Since the publication of the 2023 ESC ACS guidelines, multiple studies and an ESC/EAS dyslipidaemia update have refined how clinicians diagnose, revascularize, and treat ACS across the care continuum. **Content:** This state-of-the-art review synthesizes advances from 2023 to 2025 across five domains. Diagnosis: High-sensitivity troponin-based accelerated pathways remain foundational; GRACE 3.0 improves calibration for early vs. delayed angiography, while selective use of CCTA and routine use of intracoronary imaging/physiology help define the mechanism and optimize PCI. Revascularization: complete revascularization continues to underpin care in multivessel disease, with recent data favouring culprit-only PCI acutely and staged non-culprit treatment during the index stay in most STEMI presentations, particularly with heart-failure physiology. Antithrombotic therapy: Aspirin remains critical early after ACS-PCI; emerging evidence supports shorter DAPT and aspirin withdrawal after 1 month in carefully selected, low-ischaemic-risk patients, whereas day-0 aspirin-free strategies in unselected ACS are not non-inferior. Secondary prevention: A “strike early and strong” approach to LDL-cholesterol—often with combination therapy in hospital—is emphasized, alongside nuanced roles for SGLT2 inhibitors and GLP-1 receptor agonists. Special populations and implementation: Sex- and age-aware tailoring (including MINOCA/SCAD evaluation), pragmatic bleeding-risk mitigation, digitally enabled cardiac rehabilitation, and registry-driven quality improvement translate evidence into practice. **Summary:** Contemporary ACS care is moving from uniform protocols toward risk-stratified, mechanism-informed pathways. We offer practical algorithms and checklists to align interventional timing, antithrombotic intensity/duration, and secondary prevention with individual patient risk—bridging new evidence to bedside decisions.

## 1. Introduction

Acute coronary syndromes (ACSs) remain a leading global health challenge, with enormous clinical and societal impact. Cardiovascular disease is still a primary cause of death, accounting for nearly 17.9 million deaths annually worldwide with acute coronary events comprising a significant proportion of this burden [1]. For example, in the United States alone, over 1.2 million people are hospitalized with an ACS per year [2]. Beyond mortality, ACS leads to substantial morbidity and healthcare costs, underscoring the importance of continually improving management strategies to reduce its toll [3].

In the attempts to standardize and improve ACS care, the 2023 ESC ACS Guidelines unified previously separate recommendations for ST-elevation myocardial infarction (STEMI) and non-ST-elevation ACS into a single document covering the entire spectrum of ACSs, from unstable angina through NSTEMI to STEMI [4]. This consolidated guideline covers the spectrum from initial diagnosis and risk stratification at presentation to acute treatment and long-term secondary prevention [5]. It core management components are emphasized from prompt invasive assessment and revascularization when indicated, to appropriate anti-thrombotic therapy, and organized transition to secondary prevention with a strong focus on patient-centred care [5]. These 2023 guidelines essentially reinforced the existing evidence-based framework for ACS management while addressing nuanced updates across various aspects of care.

However, the evidence base for ACS has continued to evolve since 2023. The year 2025 marks a pivotal juncture, bringing forth new clinical trial data and focused guideline refinements with practical impacts. Several high-impact trials reported in 2025 have challenged conventional approaches and informed refinements in care. For example, studies investigating early aspirin withdrawal after PCI in ACS patients and ultra-short dual antiplatelet therapy (DAPT) regimens have yielded valuable insights into balancing ischaemic and bleeding risks [6,7]. In parallel, a focused ESC/EAS dyslipidaemia guideline update was issued in 2025 to integrate new evidence in lipid management advocating more aggressive cholesterol lowering during the index ACS hospitalization (including combination therapy with high-intensity statins and ezetimibe) for eligible patients; moreover, emerging therapeutics for high-risk individuals (such as bempedoic acid for statin-intolerant patients) are introduced [5]. Together, these developments signal further evolution of ACS management beyond the state of practice codified in 2023.

Several key thematic shifts in clinical strategy have gained momentum since the last guideline publication in 2023. First, in the context of aspirin-sparing antithrombotic regimens, recent trials suggest that dropping aspirin early (after an initial period on DAPT) and continuing P2Y-inhibitor monotherapy can reduce bleeding without increasing ischaemic events in carefully selected patients. A second major theme is individualizing DAPT duration based on patient risk profiles and individual scores to tailor therapy length, allowing shortened DAPT courses for those at high bleeding risk and prolonged therapy for those at high thrombotic risk [4]. Third, the timing of invasive interventions in NSTE-ACS is becoming more personalized; rather than a fixed schedule for all, the urgency of angiography/PCI is guided by risk stratification with an immediate or <24 h invasive strategy for very-high-risk features, versus 24–72 h for intermediate risk, and a delayed or conservative approach for low-risk clinical features [8]. Forth, lipid management is being optimized with recommendations for earlier and intensive LDL-cholesterol lowering after ACS; high-intensity statins plus ezetimibe and the use of novel agents (PCSK9 inhibitors, bempedoic acid) may be initiated at the index hospital admission to achieve stringent LDL targets [9]. Finally, secondary prevention is increasingly tailored to individual patient profiles customizing post-ACS therapy to comorbid conditions and residual risk factors (for example, selecting glucose-lowering agents with proven cardiovascular benefits in patients with diabetes, adding anti-inflammatory therapy such as low-dose colchicine in appropriate high-risk cases [5], and ensuring holistic risk factor management through lifestyle and diet, rehabilitation, and patient engagement). These thematic shifts, which will be explored in detail in subsequent sections, represent a move toward more personalized, risk-adjusted care in ACS.

In this review, new findings and recommendations are fused into a risk-stratified approach to key clinical decisions by the frontline clinician from acute reperfusion and antithrombotic therapy to lipid control and long-term prevention, providing clear, evidence-informed strategies that clinicians can apply at the bedside. By distilling the 2023–2025 developments into actionable recommendations, this review seeks to support clinicians in delivering the most current, personalized care for patients with ACS [10]. Recent important clinical trials are summarized in Table 1.

## 2. Pathophysiology and Evolving Phenotypes of ACS

ACSs arise predominantly from atherothrombotic plaque disruption in epicardial coronary arteries, triggering platelet activation and fibrin-rich thrombus with downstream myocardial ischaemia and necrosis. The Fourth Universal Definition of Myocardial Infarction (UDMI) distinguishes Type 1 MI (primary atherothrombosis from plaque rupture, erosion, or calcified nodule) from Type 2 MI (supply–demand mismatch without acute plaque disruption) and from acute or chronic myocardial injury due to non-ischaemic causes [5,11].

### 2.1. Plaque Disruption Phenotypes: Rupture, Erosion, and Calcified Nodule

Histopathology and intracoronary imaging have shown that plaque rupture (thin-cap fibroatheroma fissuring with exposure of lipid core) remains the commonest substrate in ACS, while plaque erosion (endothelial denudation over proteoglycan-rich plaque without cap rupture) and calcified nodules (eruptive calcific protrusions through a disrupted cap) are much less frequent causes of ACS but may carry distinct clinical implications. Optical coherence tomography (OCT) has enabled in-vivo discrimination of these substrates and has catalysed trials of stent-less management for carefully selected erosion lesions. In the prospective EROSION study, ACS patients with OCT-defined erosion treated with intensive antithrombotic therapy without stenting showed high 1-year event-free survival and progressive thrombus resolution on serial OCT; subsequent reports describe durable outcomes at longer follow-ups and clinical predictors favouring a non-stenting strategy [12].

Conversely, calcified nodules have been recognized as a high-risk substrate in ACS and PCI, associated with procedural complexity and adverse outcomes; mechanistic data suggest thrombosis may be initiated by fragmentation of necrotic-core calcifications with cap disruption. These observations argue for routine use of intravascular imaging to optimize PCI (lesion preparation, stent expansion) and, where appropriate, to consider conservative strategies in erosion but not in nodular calcification [13,14,15].

### 2.2. MINOCA: Myocardial Infarction with Non-Obstructive Coronary Arteries

MINOCA (MI with less than 50% angiographic lumen reduction) represents 5–10% of MIs and is a working diagnosis encompassing multiple mechanisms ranging from plaque disruption with distal embolization to coronary spasm, coronary microvascular dysfunction, spontaneous coronary artery dissection (SCAD), and non-ischaemic mimics (myocarditis, takotsubo). Contemporary position papers emphasize systematic cardiac imaging including CMR to differentiate myocarditis/takotsubo and coronary testing for vasoreactivity or intracoronary imaging to identify the culprit endotype for potential focal therapy [16,17].

### 2.3. SCAD: An Under-Recognized Cause of ACS

Spontaneous coronary artery dissection (SCAD) is understood as non-atherosclerotic, non-iatrogenic separation of the coronary wall due to intimal tear or intramural haematoma. SCAD is an increasingly recognized cause of ACS, particularly in younger women and often associated with fibromuscular dysplasia or the peri-/post-partum state. The prevailing paradigm is conservative management in a haemodynamically stable patient, given high rates of spontaneous healing and the technical hazards of wiring for PCI in dissected segments. Invasive therapy is reserved for ongoing ischaemia or left main/proximal critical flow limitation [18,19].

### 2.4. Coronary Spasm and Microvascular Dysfunction: Vasomotor Phenotypes in ACS

Epicardial coronary vasospasm can precipitate ACS through transient, intense vasoconstriction. International COVADIS criteria have standardized the diagnosis of vasospastic angina, while parallel consensus documents define microvascular angina (MVA) due to coronary microvascular dysfunction (CMD) a mechanism that can often coexist with epicardial disease and is likely to contribute to MINOCA presentations. Recognition of these vasomotor phenotypes is clinically relevant because treatment (calcium-channel blockers and nitrates for spasm; tailored anti-anginal and risk-factor therapy for CMD) differs from purely atherothrombotic strategies and is associated with improved outcomes when targeted appropriately [20,21,22].

### 2.5. Immuno-Thrombosis and Thrombo-Inflammatory Milieu

Beyond structural plaque pathology, thrombosis triggered by an activated immune system including formation of neutrophil extracellular traps (NETs) appears to amplify coronary thrombosis and subsequent myocardial injury presenting as ACS. NETs provide a scaffold for platelets and coagulation factors and have been identified within human coronary thrombi; emerging translational work links modulation of inflammatory pathways to reductions in circulating NETs and myocardial injury, underscoring the biologic rationale for anti-inflammatory strategies in secondary prevention. While translational, these data complement clinical evidence for inflammation as a residual-risk driver after ACS [23,24,25].

Mounting evidence places the IL-1β-IL-6-CRP axis at the centre of residual inflammatory risk after MI. Targeted upstream blockades have shown signals of benefit (e.g., canakinumab reducing recurrent events post-MI; tocilizumab increasing myocardial salvage in STEMI without definitively shrinking final infarct size), and inclacumab attenuated peri-procedural injury in NSTEMI. By contrast, broader/downstream inhibitors (sPLA_2_, Lp-PLA_2_, p38-MAPK) and low-dose methotrexate have been neutral or harmful, while low-dose colchicine consistently reduces events in secondary prevention. Clinically, anti-inflammatory therapy in ACS is not a class effect: the benefit likely concentrates in patients with residual inflammatory risk and depends on timing and supporting phenotype- and time-sensitive strategies [25].

## 3. Current Diagnostics of ACS

Building on established 0/1 h and 0/2 h hs-troponin pathways and GRACE-based risk triage, 2025 updates refine where non-invasive CCTA and invasive imaging add value and highlight the calibration gains of GRACE 3.0 for timing decisions in NSTE-ACS.

### 3.1. Biomarkers and Accelerated Pathways

The introduction of high-sensitivity cardiac troponins (hs-cTn) has revolutionised ACS diagnostic work-up, with earlier detection of myocardial injury at lower thresholds. Contemporary ESC guidelines endorse accelerated diagnostic algorithms such as the 0–1 h and 0–2 h hs-cTn protocols with the goal to rule out or confirm myocardial infarction in the emergency department [5]. These strategies enable rapid triaging, reduce unnecessary admissions, and are likely to support early initiation of guideline-directed therapy. Large real-world validation studies have confirmed that accelerated hs-cTn pathways achieve negative predictive values > 99%, leading to reduced length of stay and no compromise in safety. Importantly, clinicians must interpret troponin changes in clinical context as dynamic troponin changes may also reflect Type 2 MI (supply–demand mismatch) or non-ischaemic myocardial injury, which requires different management approaches.

### 3.2. Clinical Risk Stratification

Accurate risk stratification is essential to tailor diagnostic intensity and timing of invasive evaluation. The GRACE score has been the cornerstone of risk prediction in NSTE-ACS; in 2025, the newly validated GRACE 3.0 model provided improved calibration across sexes and age groups, incorporating contemporary cohorts [26]. GRACE 3.0 retains high discriminative ability for predicting death and MI and is expected to support more individualized decisions regarding early vs. delayed angiography [27]. At ESC 2025, discussions also highlighted the emerging role of machine learning and AI-enhanced risk models, which may outperform traditional scores in selected settings, though integration into clinical practice is still evolving [28].

### 3.3. Non-Invasive Imaging

Coronary CT angiography (CCTA) has become increasingly valuable for the rapid rule-out of obstructive CAD in patients with low-to-intermediate pre-test probability of ACS; the ESC guidelines recommend its use in patients with suspected NSTE-ACS when the diagnosis remains uncertain after biomarker and ECG evaluation. The recent PULSE trial tested routine 6-month CCTA surveillance after left-main PCI and reported that spontaneous MI rates trended numerically lower with scheduled CT, but the strategy did not reduce the composite endpoint of death, MI, unstable angina, or stent thrombosis compared with symptom-driven follow-up [29]. These findings argue against routine blanket imaging and support a selective approach, reserving follow-up CT for complex anatomies or unexplained symptoms.

### 3.4. Invasive Imaging and Coronary Physiology

Intravascular ultrasound (IVUS) and optical coherence tomography (OCT) provide mechanistic insights into ACS lesions, identifying plaque rupture, erosion, or calcified nodules, and guiding PCI optimization [30,31,32]. While no single pivotal RCT in 2025 changed guideline recommendations, pooled evidence and large registries consistently show that imaging-guided PCI improves stent expansion and reduces stent thrombosis and target-lesion failure, particularly in complex lesions [33]. Fractional flow reserve (FFR) and instantaneous wave-free ratio (iFR) also continue to refine lesion selection in multivessel disease [34]. The growing emphasis is on integrating invasive imaging and physiology not just to improve procedural results but also to enhance diagnostic accuracy in ACS phenotypes (e.g., distinguishing plaque erosion amenable to conservative management).

## 4. Invasive Management of ACS

Prior trials established the benefit of complete revascularization in multivessel STEMI; 2025 data add nuances on when to address non-culprit lesions and how imaging should guide PCI optimization.

### 4.1. Culprit-Only Versus Complete Revascularization in STEMI

For patients with STEMI and multivessel disease, complete revascularization improves outcomes versus culprit-only PCI established by the pivotal COMPLETE trial in 2019, which demonstrated reductions in cardiovascular death or MI when non-culprit lesions were treated during the index admission or soon thereafter [35,36]. Building on this foundation and providing more granular insight, the OPTION-STEMI trial [37] randomized 994 STEMI patients to immediate complete revascularization in the index procedure versus staged complete revascularization later during the same hospitalization; it concluded that at 1 year, the primary composite of death/MI/unplanned revascularization was 13.1% with immediate complete revascularization and thus higher than 10.8% with staged PCI, and noninferiority of immediate complete revascularization could not be demonstrated (*p* for noninferiority = 0.24). In fact, a prespecified subgroup showed harm with immediate complete revascularization, e.g., in those patients presenting with signs of heart failure (Killip ≥ II). This very recent data support culprit-only PCI acutely followed by staged non-culprit PCI prior to hospital discharge for most patients, especially when haemodynamic compromise or heart-failure symptoms are present.

### 4.2. Role of Intracoronary Imaging and Coronary Physiology

**Figure 1:**
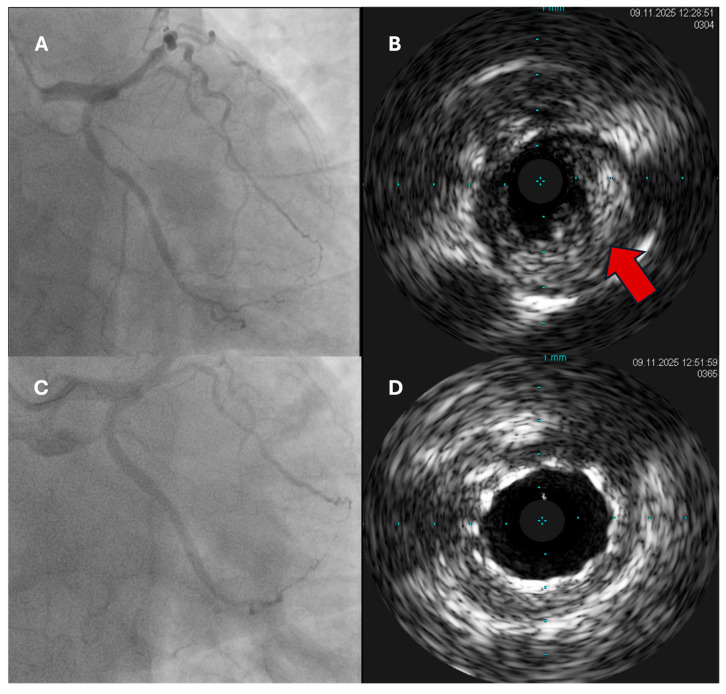
An example of IVUS application in a PPCI for ACS case. (**A**) Diagnostic coronary angiogram shows LAD culprit lesion; (**B**) IVUS shows the culprit lesion with a large soft plaque (arrow) compressing lumen; (**C**) successful PCI to LAD lesion with a drug eluting stent; (**D**) IVUS shows fully opened lumen and deployed stent.

**Figure 2:**
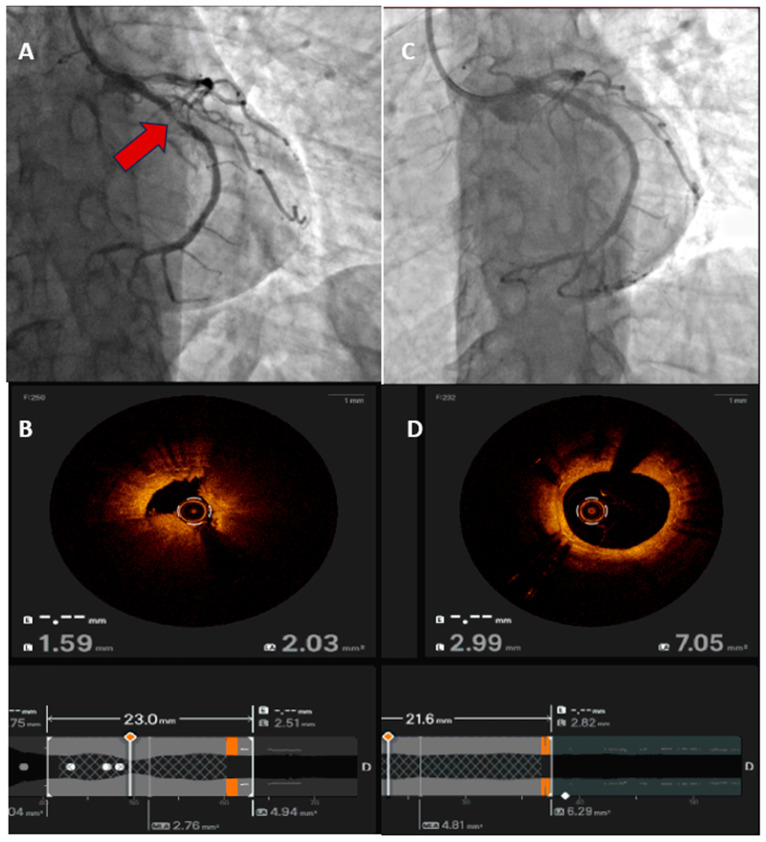
An example of OCT-guided ACS intervention. (**A**) Angiogram shows a significant stenosis (arrow) at proximal LCX; (**B**) OCT shows ruptured plague and lumen condition; (**C**) angiogram result after PCI to culprit lesion; (**D**) OCT shows the result after stent deployed.

Intravascular imaging (IVUS/OCT) has become central to PCI optimization in ACS, improving stent sizing, expansion, and apposition, and reducing adverse events in complex lesions (Figure 1). A large contemporary meta-analysis reported significantly lower composite adverse outcomes with imaging-guided versus angiography-guided stenting, corroborating earlier pooled data linking imaging guidance to improved clinical endpoints [38] (Figure 2). Although physiology (FFR/iFR) is widely used to assess non-culprit lesions, the FLOWER-MI RCT showed no superiority of FFR-guided over angiography-guided complete revascularization in STEMI, underscoring that physiology is helpful for lesion selection but not universally outcome-superior in the acute MI setting [34]. In practice, 2025 trends favour routine imaging guidance for ACS PCI (especially left main/bifurcation/calcified disease) and selective use of physiology to refine decisions on non-culprit lesions once the patient is stable [38].

### 4.3. Post-PCI Surveillance Imaging

Routine anatomic surveillance after PCI is not supported by new randomized evidence. In PULSE (*n* = 606), routine 6-month CCTA did not lower the 18-month composite of all-cause death, spontaneous MI, unstable angina, or stent thrombosis versus symptom/ischaemia-driven follow-up. Although spontaneous MI was reduced with routine CCTA, this came with more imaging-triggered revascularization and no difference in clinically driven TLR—arguing against routine surveillance and in favour of selective CCTA (e.g., complex anatomy or unexplained symptoms), given added radiation/iodine exposure, clinic time, and procedure cascades. These findings argue against unselected CCTA for surveillance, favouring follow-up CCTA imaging only in anatomically complex cases or when symptoms/tests raise concern [29,39]. Accordingly, PULSE’s negative primary, reduction in spontaneous MI, and increase in imaging-triggered revascularization should be viewed as hypothesis-generating for selected subsets, but not a mandate for routine CCTA.

## 5. Antithrombotic Therapy After ACS

Twelve months of DAPT remains the historical default after ACS-PCI; recent trials explore who may safely shorten therapy and when aspirin can be withdrawn without trading off early ischaemic protection.

### 5.1. Acute-Phase and Peri-Procedural Considerations (Including Shock)

Aspirin plus a potent P2Y_12_ inhibitor (prasugrel or ticagrelor) remains the default antiplatelet backbone for ACS patients undergoing PCI, with prasugrel preferred over ticagrelor in invasive pathways per ESC 2023 guidance; clopidogrel is reserved for intolerance or where potent agents are unsuitable [5]. In cardiogenic-shock AMI, the DAPT-SHOCK AMI randomized trial addressed the practical problem of unreliable oral absorption; early intravenous Cangrelor administration achieved universal platelet inhibition at the end of primary PCI (100% compared to 22% with crushed oral ticagrelor) without excess major bleeding complications. However, Cangrelor did not meet noninferiority criteria to oral crushed ticagrelor for the 30-day composite of death/MI/stroke (37.6% vs. 41.0%; difference—3.5%; 95% CI −11.2 to 4.3; *p* for noninferiority = 0.13). Signals favoured Cangrelor for several secondary outcomes and 12-month mortality, supporting its use as a bridging strategy when enteral uptake is doubtful, with timely transition to an oral agent once stable [40].

### 5.2. DAPT Composition and Duration After PCI and Tailored De-Escalation

While the ESC 2023 guidelines recommended 12 months of DAPT (aspirin + potent P2Y_12_ inhibitor) after ACS-PCI, it offered some room for shorter duration of DAPT in patients at high bleeding risk and a step-down to single antiplatelet therapy only in event-free, lower-risk patients [5]. Over the last 3 years, new scientific evidence has emerged to address some scenarios in patients post-PCI for ACS in more detail and with a view to lower the risk of bleeding.

(a) “Immediate” aspirin-free strategy (day 0–4): NEO-MINDSET.

In 3410 ACS patients randomized within 4 days post-PCI to P2Y_12_ monotherapy (ticagrelor/prasugrel) vs. 12-month DAPT, noninferiority was not demonstrated for the ischaemic composite at 12 months (7.0% vs. 5.5%; HR 1.28, 95% CI 0.98–1.68); however, a landmark analysis localized an excess ischaemic risk within the first 30 days, while bleeding was lower with monotherapy at both 30 days and 12 months, with the conclusion, at least in unselected ACS cases soon after PCI, to keep aspirin on board in the early weeks [7].

(b) Early aspirin discontinuation after 1 month in selected low-risk MI: TARGET-FIRST.

In MI patients with complete revascularization, event-free at 1 month on DAPT, switching to 11 months of P2Y_12_ monotherapy was noninferior to continued DAPT for net cardiovascular/cerebrovascular outcomes and reduced clinically relevant bleeding (BARC 2/3/5 2.65% vs. 5.57%, HR 0.46; 95% CI 0.29–0.75; *p* = 0.002). This supports targeted de-escalation after 1 month in carefully profiled, low-ischaemic-risk MI [6].

(c) Short-course DAPT in all-comers: DUAL-ACS.

A pragmatic, all-comer MI trial comparing 3 vs. 12 months of DAPT found similar ischaemic outcomes, with numerically lower all-cause mortality (2.7% vs. 3.4%) and less major bleeding (3.2% vs. 4.0%) at 15 months with the 3-month strategy thereby poised to individualized shortening beyond high-bleeding-risk cohorts. (Underpowered for strict noninferiority, but directionally consistent with prior data [41].)

(d) Complex/high-risk PCI: avoid “yo-yo” intensity—TAILORED-CHIP.

In patients with complex anatomy or high ischaemic risk, a tailored regimen (early escalation to low-dose ticagrelor + aspirin, then late de-escalation to clopidogrel monotherapy) failed to improve net outcomes versus standard DAPT and increased clinically relevant bleeding (7.2% vs. 4.8%). Standard DAPT remains appropriate; avoid unnecessary early intensification [42].

In NEO-MINDSET, potent P2Y_12_-inhibitor monotherapy initiated within 4 days of ACS-PCI failed to meet noninferiority for the ischaemic composite vs. 12-month DAPT, with an early (≤30 days) hazard signal and more stent thrombosis, despite less bleeding cautioning against day-0 aspirin withdrawal outside highly selected contexts. Conversely, the pragmatic DUAL-ACS trial suggests 3 months of DAPT may achieve similar ischaemic outcomes with less major bleeding versus 12 months, but there is critique because of lower-than-expected event rates and an open-label design limiting definitive conclusions. Thus, the use of 3 months of DAPT should be individualized to patients with lower ischaemic and higher bleeding risk.

### 5.3. CABG After ACS

Two recent trials have refined antiplatelet medication after surgery; TACSI (ACS-CABG) showed that ticagrelor + aspirin for 12 months did not reduce death/MI/stroke versus aspirin alone but increased major bleeding (4.9% vs. 2.0%). Aspirin monotherapy is therefore an appropriate default therapy after ACS-CABG [43].

The TOP-CABG study focussed on a de-escalating approach (DAPT for 3 months then aspirin alone for 12 months) and demonstrated a noninferior rate of saphenous vein graft occlusion with the de-escalating strategy as compared to 12-month DAPD; in addition, there was less clinically relevant bleeding supporting short DAPT followed by aspirin alone as the recommended best trade-off between vein graft patency and bleeding risks [44].

### 5.4. Patients Who Also Require Oral Anticoagulation

For ACS patients with an indication for long-term oral anticoagulation (such as atrial fibrillation), the 2023 ESC guidance recommended very short triple therapy (OAC + aspirin + clopidogrel) for up to 1 week (extended to 1 month in very high ischaemic risk scenarios), followed by dual therapy (OAC + a single antiplatelet, preferably clopidogrel) for 12 months, followed by OAC alone. These principles still stand in 2025 and are aligned with contemporary evidence [45].

### 5.5. Gastrointestinal Bleeding Prevention During Antithrombotic Therapy

Proton-pump inhibitor (PPI) co-therapy is recommended for patients at high GI-bleeding risk receiving DAPT or triple therapy; risk tools (e.g., PRECISE-DAPT, clinical history of ulcer/bleed, anaemia, advanced age) can guide routine prescription. Against this backdrop, the nationwide cluster-randomized HELP-MI trial found that routine Helicobacter pylori screening/eradication did not significantly reduce upper-GI bleeding at 1 year overall; any benefit appears concentrated in selected high-risk phenotypes (e.g., anaemia). Thus, PPI co-therapy remains first-line, with targeted H. pylori testing considered for very high-risk patients or those with prior ulcer disease.

Routine Helicobacter pylori screening during MI hospitalization did not significantly reduce upper-GI bleeding in the nationwide cluster-randomized HELP-MI SWEDEHEART trial (18,466 patients; rate ratio 0.90; 95% CI 0.77–1.05) despite the observation that subgroups of patients with anaemia may potentially benefit. Therefore, targeted screening for high-bleeding-risk phenotypes may be reasonable while routine universal screening is not supported [46].

### 5.6. Where Do Factor XI/XIa Inhibitors Fit?

The phase II PACIFIC-AMI trial showed potent FXIa suppression without an overt bleeding penalty when added to DAPT after MI, whereas clinical observational data from the AF mega-trial OCEANIC-AF demonstrated inferior stroke prevention with asundexian versus apixaban, tempering immediate enthusiasm. ACS-specific phase III programmes (milvexian) are ongoing; there is no practice-changing role yet in ACS secondary prevention [47].

## 6. Secondary Prevention and Long-Term Care

Aggressive lipid-lowering, neurohormonal therapy, smoking cessation, and cardiac rehabilitation remain the pillars of long-term care; current guidance emphasizes earlier-combination lipid therapy, and contemporary trials clarify the roles of SGLT2i and GLP-1RA.

### 6.1. Lipid-Lowering Strategies and Residual Cardiovascular Risk

Recurrent ischaemic risk after ACS remains front-loaded in the first months, with substantial cumulative risk thereafter; consequently, the 2025 ESC/EAS Focused Update reframes lipid management around earlier and more intensive therapy during the index hospitalization (“the sooner, the lower, the better”). The document keeps the numeric LDL-C goals from 2019—at least a 50% reduction from baseline and <1.4 mmol/L (<55 mg/dL) for very-high-risk secondary prevention, with <1.0 mmol/L (<40 mg/dL) considered after a second vascular event within 2 years, while newly emphasizing immediate combination therapy (high-intensity statin plus ezetimibe, and rapid addition of a PCSK9 pathway agent when needed) rather than a slow, stepwise escalation after discharge. It also upgraded guidance on once-in-a-lifetime lipoprotein (a) testing (risk begins to rise above ~30 mg/dL [~62 nmol/L] and is clearly higher at ≥50 mg/dL [≥ 105 nmol/L]) and clarifies triglyceride-targeted therapy [48,49].

The update reports imaging and clinical evidence that very early, deep LDL-C lowering is feasible and correlates with favourable plaque modification (HUYGENS, PACMAN-AMI) and earlier goal attainment than a statin-alone approach. It recommends initiating high-intensity statin on day 0 and planning combination therapy before discharge if the predicted LDL-C will not meet the target, rather than waiting for delayed outpatient titration [48].

For hypertriglyceridemia on top of statin therapy, the update distinguishes icosapent ethyl (EPA ethyl ester), which reduced events in REDUCE-IT, from mixed EPA/DHA omega-3 formulations that were neutral in STRENGTH; when fasting TGs are ~135–499 mg/dL despite statins, high-dose icosapent ethyl should be considered [48,50,51]. In statin-intolerant patients, bempedoic acid lowered MACE in CLEAR outcomes and is now integrated into the ESC/EAS treatment ladder [9]. Inclisiran offers durable ~50% LDL-C lowering with twice-yearly maintenance and is highlighted as an adherence-friendly option while we await definitive outcomes [52,53].

Finally, CABG-specific secondary prevention data matured in 2025: despite ~48% placebo-adjusted LDL-C-lowering, NEWTON-CABG (CardioLink-5) found no reduction in saphenous vein graft disease at 2 years with evolocumab versus placebo, underscoring that early graft failure mechanisms extend beyond atherothrombosis [54].

### 6.2. Neurohormonal Blockade and Blood Pressure Targets

Decades of randomized evidence support ACE inhibitors/ARBs and mineralocorticoid receptor antagonists in post-MI patients—especially when LV dysfunction or heart failure is present (SAVE, VALIANT, HOPE, EPHESUS). These therapies reduce mortality and recurrent ischaemic events and remain foundational unless contraindicated [55,56,57].

The role of long-term beta-blockers is being refined. The multicentre REBOOT trial (2025) showed no significant reduction in its primary composite outcome with routine beta-blocker therapy in patients without heart failure after MI, aligning with contemporary registry analyses and smaller RCTs that question blanket, indefinite use when LVEF is preserved and revascularization complete. Beta-blockers remain indicated for angina control, arrhythmia suppression, and when LVEF is reduced or heart failure is present [58].

Contemporary RCTs diverge on the use of beta-blockers: REBOOT (LVEF > 40%) showed no overall benefit and a potential signal of harm in women with normal EF, whereas BETAMI–DANBLOCK (LVEF ≥ 40%) found a modest reduction in a broad composite endpoint, mainly via fewer MI. Interestingly, pooled patient-level data demonstrate some benefit when LVEF is 40–49% and neutrality when ≥50%. Clinically, continued β-blockers are recommended when LVEF ≤ 40% or when there are other indications (angina, arrhythmia, hypertension); consider 6–12 months when LVEF is 40–49%, and avoid routine long-term therapy when LVEF is ≥50% with re-evaluation at 6–12 weeks.

In practice, continue β-blockers indefinitely if LVEF is ≤40% or another indication exists (angina, arrhythmia, hypertension). When LVEF is 40–49%, consider 6–12-month therapy and reassess; when LVEF is ≥50%, avoid routine long-term therapy and reassess at 6–12 weeks, particularly in women or those with bradycardia, hypotension, or fatigue.

### 6.3. Cardiometabolic Drugs SGLT2 Inhibitors and GLP-1 Receptor Agonists

Two large trials tested early SGLT2 initiation after MI. EMPACT-MI randomized patients within 14 days of MI enriched for HF risk (LVEF < 45% and/or congestion); the primary composite of all-cause death or first hospitalization for heart failure (HF) was neutral (HR 0.90), but HF hospitalizations were reduced (HR ≈ 0.77) [59]. DAPA-MI, by contrast, enrolled patients without diabetes or chronic HF and adopted a hierarchical cardiometabolic composite because of low event rates. The win ratio favoured dapagliflozin (1.34), driven by metabolic benefits, with no reduction in classic CV events over ~1 year [60]. Together, these trials support safety and HF-related benefits but do not establish MACE reduction for early, universal SGLT2 use post-MI; targeted initiation in HF-prone phenotypes is reasonable pending MACE-powered data. Taken together, early SGLT2 therapy appears safe post-MI and favourable for metabolic recovery and HF-related surrogates, but a reduction in post-MI hard endpoints remains to be proven. In contrast, weekly semaglutide (2.4 mg) in a pure secondary-prevention cohort with overweight/obesity (many with prior MI) reduced 3-point MACE by 20% (HR 0.80) in the SELECT trial, providing a cardioprotective option for eligible ACS survivors, integrated with lifestyle and lipid-lowering therapy [61].

### 6.4. Comprehensive Cardiovascular Prevention Beyond Medication

Cardiac rehabilitation (CR) remains among the highest-value therapies in secondary prevention. A 2023 meta-analysis of 85 RCTs (*n* ≈ 23,400) showed significant reductions in CV mortality (RR 0.74), hospitalizations (RR 0.77), and MIs (RR 0.82), with numbers-needed-to-treat ~37–100.19. Contemporary cohort data and program innovations (home-based and hybrid models) reinforce improved survival and risk-factor control, and digitally enabled CR national programmes have demonstrated meaningful population-level risk-factor improvements [62,63].

Smoking cessation is imperative; contemporary data dispel any “smoker’s paradox” after STEMI and emphasize worse adjusted outcomes in smokers. Evidence-based pharmacotherapy should be offered to every tobacco user: high-certainty Cochrane evidence shows combination NRT (patch + rapid-acting form) improves long-term abstinence vs. single-form NRT, and network meta-analyses identify varenicline as among the most effective monotherapies [64,65,66].

Improving adherence and simplifying regimens also reduces events. In SECURE, a fixed-dose polypill (aspirin + ramipril + atorvastatin) after recent MI lowered the primary composite of CV death, non-fatal MI, non-fatal stroke, or urgent revascularization (HR 0.76) and reduced CV death by 33%, with better self-reported adherence [67]. Finally, influenza vaccination soon after MI reduced a composite of death/MI/stent thrombosis (HR 0.72) and both all-cause and CV mortality at 12 months in the double-blind IAMI RCT; vaccination should be embedded into post-MI care pathways [68].

Beyond the standard ACS patient, several groups feature prognostically relevant differences. Recently, guideline and trial updates have sharpened gender-specific antithrombotic choices, clarified management in the elderly/frail, updated pregnancy care, and reinforced risk-tailoring in chronic kidney disease (CKD), alongside attention to racial/ethnic and socioeconomic disparities in access to care and outcomes [5].

### 6.5. Female Patients with ACS

Women continue to face delays in recognition, lower use of guideline therapies, and worse adjusted outcomes after ACS across multiple health systems. Contemporary registry analyses document higher in-hospital and long-term adverse events in women, with underuse of early DAPT and reperfusion compared to men. These disparities persist despite adjustment for age and comorbidities, highlighting gaps in systems of care and implementation [69,70].

Female patients more often present with non-obstructive disease, single-vessel involvement, and non-rupture phenotypes (e.g., plaque erosion), factors that may modify invasive and antithrombotic decisions. MINOCA is disproportionately prevalent in women (≈6–15% of MIs overall; up to ~50% of MINOCA cohorts), underscoring the value of intravascular imaging and CMR to define mechanisms (plaque disruption with distal embolization, vasospasm, SCAD, or microvascular dysfunction) [71,72,73].

The 2025 EAPCI/ESC Working Group on Thrombosis consensus provides practical, gender-specific, antithrombotic guidance, recognizing higher bleeding risk in women (older age, lower body weight, CKD), and emphasizes radial access to reduce access-site bleeding and individually tailor DAPT intensity/duration by virtue of validated bleeding risk prediction tools (PRECISE-DAPT; ARC-HBR) rather than applying default regimens. It also draws attention to female under-representation in trials and recommends that ongoing studies prespecify sex-stratified analyses [74]. For bleeding risk stratification, the PRECISE-DAPT score (five items) and ARC-HBR criteria remain the reference frameworks to individualize DAPT duration and intensity [75,76].

### 6.6. Older Adults and Patients Living with Frailty

Evidence for invasive management in older patients is more nuanced. The SENIOR-RITA randomized trial (*n* = 1518; ≥75 years, NSTEMI) found that a routine invasive strategy did not reduce the primary composite of cardiovascular death or non-fatal MI over a median of 4.1 years versus a conservative approach; although non-fatal MI was lower with an invasive strategy, bleeding complications were numerically more frequent. These results support an individualized approach rather than an invasive default in advanced age [77]. Conversely, the earlier After Eighty trial (≥80 years, NSTEMI/UA) had demonstrated superiority of an invasive strategy for a composite of MI/urgent revascularization/stroke/death, emphasizing that patient selection may be the key driver to explain a beneficial effect [78]. Beyond age, frailty is a key factor as demonstrated in the MOSCA-FRAIL trial with early harm and only a late benefit of an invasive strategy in frail NSTEMI patients; the overall neutral survival effect supports the concept of shared decision making balancing short-term hazards against potential late gains [79]. Practical measures to reduce harm in older/frail adults include radial access, bleeding-aware antithrombotic selection (e.g., dose-adjusted regimens; de-escalation when appropriate), and routine use of risk/frailty instruments alongside GRACE [80].

### 6.7. Pregnancy, Postpartum, and SCAD-Predominant ACS

The 2025 ESC Pregnancy guidelines update both risk stratification and multidisciplinary care pathways for cardiovascular disease in pregnancy, including ACS. It emphasizes pre-pregnancy counselling, team-based care, medication safety tables, and SCAD-predominant mechanisms of MI in pregnancy/post-partum, where conservative management is preferred when haemodynamic parameters allow for it [81]. For SCAD, the ESC position paper provides recommendations such as the confirmation of diagnosis with intracoronary imaging when safe, no aggressive instrumentation, preference of conservative therapy with close monitoring, and reservation of PCI/CABG for cases of ongoing ischaemia, left main obstruction, or proximal critical flow limitation [82]. Recent reviews reaffirm SCAD’s predilection for younger women and some association with fibromuscular dysplasia [83].

### 6.8. Chronic Kidney Disease (CKD)

Presence of CKD increases both ischaemic and bleeding risk; severe CKD (eGFR < 30 mL/min/1.73 m^2^) is an ARC-HBR major criterion, impacting DAPT choices and duration [76]. After an initial DAPT period, ticagrelor monotherapy (vs. continued ticagrelor + aspirin) reduced bleeding without excess ischaemia in CKD subgroups, consistent with TWILIGHT and dedicated CKD analyses and supportive of early aspirin withdrawal in selected high-bleeding-risk patients after 3 months [84,85]. Renal dose adjustment for parenteral antithrombotic agents and careful avoidance of nephrotoxins remain essential [5].

### 6.9. Ancestry-Specific Considerations (The “East Asian Paradox”)

East Asian patients often exhibit lower ischaemic and higher bleeding propensities under standard DAPT, a clinical observation summarized as the “East Asian paradox” prompting region-specific recommendations in favour of lower-intensity or shorter DAPT duration in appropriate patients and veering towards genotype- and phenotype-guided strategies. A 2025 position statement synthesizes contemporary evidence and provides pragmatic algorithms for antiplatelet selection and dosing in patients with East and South Asian heritage [86].

### 6.10. Cardiogenic Shock (CS)

Present in 5–8% of ACSs, CS represents a state of critical circulatory failure in which the heart is unable to maintain sufficient tissue perfusion, leading to multiorgan dysfunction and high mortality of up to 50% [5]. Temporary mechanical circulatory support (MCS) devices have emerged as essential tools to stabilize haemodynamics, serving as a bridge to recovery, a bridge to decision, or a bridge to more definitive therapies such as durable ventricular assist devices or heart transplantation. Despite technological advances, optimal timing, patient selection, and device strategy remain controversial and are largely guided by observational data and expert consensus rather than robust randomized evidence. The main temporary MCS modalities include the intra-aortic balloon pump (IABP), veno-arterial extracorporeal membrane oxygenation (V-A ECMO), and microaxial flow pump. The decision to implement MCS depends on haemodynamic status, the reversibility of the underlying cause, and patient factors such as age, frailty, and comorbidities. In AMI-CS, early identification of low-flow states and prompt revascularization remain central. Landmark trials illustrate that while IABP (IABP-Shock II) and ECMO (ECLS-Shock) do not improve survival [85,86], the DanGer-Shock trial found that early implantation of the Impella CP in patients with predominant left ventricular failure due to STEMI significantly reduced six-month as well as long-term mortality, albeit with higher complication rates including bleeding, ischemia, and sepsis [87,88]. The ongoing Unload-ECMO trial will compare the combination of early initiation of ECLS in combination with Impella CP as compared to ECLS alone. Current AHA guidelines recommend temporary MCS as reasonable (Class IIa, Level C) for patients with persistent hypoperfusion unresponsive to pharmacologic therapy, preferably before irreversible organ damage occurs [89]. In refractory cardiac arrest, extracorporeal cardiopulmonary resuscitation (ECPR) using V-A ECMO may be lifesaving in highly selected cases, although randomized evidence remains inconclusive [90,91,92]. However, in all MCS cases, complications remain a major limitation, including bleeding, vascular injury, haemolysis, left ventricular distension, Harlequin syndrome, and coagulopathy. These risks necessitate careful device management, anticoagulation balance, and multidisciplinary oversight through specialized shock teams.

### 6.11. Health Equity and Access

Inequities by race/ethnicity and socioeconomic position remain pronounced across the ACS continuum spanning from prehospital access to PCI-capable centres to in-hospital reperfusion times and post-discharge prescribing, all translating into heterogeneous outcomes. Recent field analyses showed that Black and Hispanic patients presenting with STEMI are less likely to present to PCI-capable hospitals and experience longer in-hospital waits for revascularization with associated mortality; systematic reviews confirm higher ACS incidence and mortality with lower SEP and lower revascularization rates [87,88,89]. These data reinforce the call for research and implementation of strategies that directly address social determinants of health and treatment standardization.

## 7. Digital Tools and Implementation

Despite major advances in diagnostics and therapeutics, a substantial fraction of preventable harm in ACS still stems from gaps in delivery including delayed or inconsistent application of guideline-directed care, incomplete secondary prevention at discharge, poor rehabilitation uptake, and inequitable follow-up. A practical way to narrow these gaps would be to embed evidence and workflows directly into clinicians’ hands and patients’ routines (via point-of-care guideline platforms, structured order sets, digitally enabled rehabilitation, and learning registries) while observing contemporary regulatory and equity guardrails [90].

The European Society of Cardiology (ESC) now distributes interactive algorithms, calculators, and risk scores through the ESC Pocket Guidelines app enabling point of care access; importantly, the app is CE-marked as medical software, underscoring its intended clinical use. In 2025, >150 digital tools exist already across 28 titles, including the consolidated ACS guidance, available on iOS/Android and desktop. In parallel, ESC Chat, an AI-assistant, returns answers exclusively from current ESC guidelines with direct citations, accelerating bedside retrieval of the relevant paragraph when time is critical. Hospitals can increase this use by installing such tools on ward devices and linking them to order sets, so that “knowing the guidelines” seamlessly becomes organising the best care.

For triaging and risk stratification, several AI-enhanced ECG analysis tools can assist emergency departments in detecting acute myocardial infarction and forecasting short-term adverse events with discrimination similar to or better than traditional pathways and clinician assessments. In a multicentre study, an AI-ECG model achieved clinically useful accuracy for AMI detection and prediction of 30-day MACE, acting as a decision-support adjunct rather than a replacement for clinical judgment [93]. Complementary machine-learning models have also been developed to stratify MACE risk among troponin-negative chest-pain patients, potentially guiding observation vs. discharge decisions. Institutions piloting such tools should pair them with explicit escalation thresholds and real-time cardiology oversight.

In the EU, most diagnostic and triage AI tools in clinical care will fall under the AI Act’s “high-risk” category, triggering obligations around risk management, post-market monitoring, transparency, and bias management; software that meets the definition of a medical device must also comply with the Medical Device Regulation (MDR). Practical guidance for Medical Device Software (including AI) clarifies qualification and classification criteria and expectations for placing such software on the market. In short, health care systems should treat AI-enabled decision support like any other device by maintaining a model inventory, verifying local performance, and monitoring equity and safety outcomes over time.

Digitally structured discharge and medicine optimization can hard-wire guideline-conforming therapies and recommendations at discharge. For example, a fixed-dose polypill strategy (aspirin/ramipril/atorvastatin) reduced recurrent events and cardiovascular mortality after MI in the randomized SECURE trial, offering an implementation-friendly route to improve adherence in routine practice. Likewise, the 2025 ESC/EAS Focused Update on dyslipidaemia recommends earlier and more intensive LDL-cholesterol lowering during the index admission to reach targets faster; the recommendation is strongly grounded in outcome trials such as IMPROVE-IT, in which adding ezetimibe to statin therapy after ACS reduced major cardiovascular events. (Very-early PCSK9-inhibitor initiation is feasible and produces rapid LDL-C reductions, though outcome data in the immediate post-ACS window remain limited.) Embedding these steps into electronic discharge pathways and defaulting to high-intensity statin plus ezetimibe when needed can establish guideline-concordant prescribing by design [67,91,92].

Analysis of digitally enabled cardiac rehabilitation (CR) has indicated that home-based or hybrid CR is at least as effective as centre-based programmes for functional gains and risk-factor control, often with higher participation when digital platforms are used. The 2023 Cochrane update and subsequent reviews support parity of clinical outcomes between home- and centre-based models, while the 2023 AHA Science Advisory offers practical standards for safe, equitable digital CR delivery. Randomized and real-world studies of mobile-app-augmented CR further suggest improvements in exercise capacity and reductions in unplanned readmissions, with effectiveness mediated by engagement quality. Given persistent capacity constraints and geographic barriers, default referral to hybrid/digital CR with remote vitals, supervised exercise modules, and structured messaging can materially expand access without sacrificing efficacy [62,94,95,96].

Learning health systems, registries, and quality indicators: Continuous feedback loops help ensure that digital tools translate into measurable improvements. The ESC’s EuroHeart programme provides standardized ACS/PCI datasets and national-level registries, with annual reports now covering tens of thousands of ACS cases across multiple countries. In 2025, the ESC published updated quality indicators (QIs) for ACS (21 measures across structures, non-invasive and invasive care, secondary prevention, and outcomes) intended for benchmarking and audit-and-feedback. Aligning local dashboards to these QIs (among them, guideline therapies at discharge, door-to-balloon times, and CR referral and completion) supports targeted Plan–Do–Study–Act cycles and complements bedside digitalization efforts [90].

Equity, access, and device bias: Digital strategies can either narrow or widen disparities. Telemedicine and remote-monitoring studies in cardiology describe persistent inequities by race/ethnicity and socioeconomic position in access and modality (video vs. phone), as well as heterogeneous digital literacy. Additionally, device-level accuracy issues, most prominently the under-recognition of hypoxaemia by pulse oximeters in darker-skinned patients and subsequent policy responses, illustrate why deployment must be coupled to ethnic and gender equity and safety monitoring. The prudent approach is to pair every rollout with an equity plan (multilingual content, offline options or loaner devices, and assisted enrolment) and to track uptake and outcomes by demographic strata, updating procurement standards as regulators (e.g., FDA) tighten performance expectations across skin tones [93,97,98].

## 8. Knowledge Gaps and Future Research

### 8.1. How Short and How “Aspirin-Free” Can We Go After ACS PCI?

Three late-breaking, randomized trials at ESC 2025 refined, but did not settle, the question. In NEO-MINDSET, stopping aspirin immediately after PCI and treating the first year with potent P2Y_12_-inhibitor monotherapy failed to meet noninferiority for ischaemic outcomes vs. standard 12-month DAPT, despite less bleeding, arguing that the first weeks remain a “DAPT-dependent” period [7]. By contrast, TARGET-FIRST (patients with low-risk MI who achieved early, complete revascularization) demonstrated that discontinuing aspirin at 1 month and continuing a potent P2Y_12_ inhibitor was noninferior to continued DAPT and reduced bleeding; the article and PubMed record were released in parallel with the ESC presentation [6]. A broader, pragmatic DUAL-ACS comparison suggested 3-month DAPT may be comparable to 12 months in all-comers but with less bleeding; given its trial design and event rates, many readers interpret these findings as hypothesis-generating and applicable mainly to carefully selected patients [41]. Taken together, these data support (i) avoidance of very-early aspirin withdrawal in unselected ACS, (ii) selective 1-month aspirin discontinuation after uncomplicated, fully revascularized MI, and (iii) a research need for risk-stratified, gender- and ethnicity-informed de-escalation frameworks rather than one-size-fits-all rules.

### 8.2. When to Complete Revascularization in Multivessel STEMI

The OPTION-STEMI RCT (simultaneously published in The Lancet) did not demonstrate noninferiority of immediate complete revascularization vs. staged complete revascularization during the same hospitalization; numerically worse outcomes were seen in patients with signs of heart failure—tempering enthusiasm for a “treat-everything-now” reflex [37]. Yet, MULTISTARS AMI (staging at ~5 weeks) found immediate multivessel PCI superior to delayed staging for stable STEMI, highlighting what we call “staged” matters: same-stay staging may differ biologically from weeks-later staging [99]. The research agenda here is to pinpoint who benefits from immediate non-culprit PCI (younger, low-LVEDP, no HF signs) and to harmonize intravascular imaging use across strategies [100].

### 8.3. Antiplatelet Therapy After CABG (Duration, Intensity, and Timing)

Two large trials unsettled long-standing assumptions. In TACSI, ticagrelor and aspirin for 12 months after CABG did not reduce death/MI/stroke/re-revascularization compared to aspirin alone, but increased major bleeding [43]. TOP-CABG proposed de-escalating to aspirin after 3 months of DAPT with similar ischaemic outcomes and less bleeding, suggesting time-limited intensification may suffice for graft protection [44]. Guideline committees will need to reconcile these signals with heterogeneity in graft types and surgical practice; high-priority trials include imaging-adjudicated graft failure endpoints with stratification by off-pump surgery and competitive flow.

### 8.4. Cardiogenic Shock and Immediate Platelet Inhibition

DAPT-SHOCK AMI was the first randomized antiplatelet trial devoted to MI with shock and showed that intravenous Cangrelor achieved immediate and potent platelet inhibition compared to crushed oral ticagrelor with no excess major bleeding; the primary noninferiority endpoint for 30-day death/MI/stroke was not met, but mortality and several secondary outcomes numerically favoured Cangrelor. Publication of the full peer-reviewed dataset is awaited; until then, centres may reasonably prefer Cangrelor for “no-oral-route/no-absorption” shock scenarios [101]. In DAPT-SHOCK AMI, noninferiority was not met for the 30-day composite vs. crushed ticagrelor despite immediate platelet inhibition and favourable secondary signals; thus, intravenous Cangrelor is reasonable as a bridge when oral uptake is doubtful, while definitive outcome benefit remains unproven.

### 8.5. Anti-Inflammatory and Anticoagulant Innovation

Low-dose colchicine reduces recurrent events after MI (COLCOT) and in chronic CAD (LoDoCo2), cementing inflammation as a modifiable pathway; however, effects in the acute MI window remain mixed, underscoring the need to balance benefit against tolerability and drug–drug interactions [102,103]. Meanwhile, enthusiasm for factor XIa inhibition as a “bleeding-sparing” strategy has cooled: PACIFIC-AMI (phase 2) added to DAPT was neutral for ischemia, and the OCEANIC-AF phase 3 programme found asundexian inferior to apixaban for stroke prevention, raising mechanistic questions about how much thrombin suppression is useful. For ACS specifically, the phase-3 LIBREXIA-ACS (milvexian) trial is ongoing and will further elucidate a class effect [47,104].

### 8.6. Glucose–Cardiorenal Agents Early After AMI

Two large trials reached different conclusions about SGLT2 inhibitors; the EMPACT-MI (empagliflozin within 14 days) was neutral on the primary composite of first HF hospitalization or all-cause death, though several HF outcomes favoured treatment. The DAPA-MI (dapagliflozin) improved cardiometabolic outcomes using a hierarchical “win ratio” but did not reduce traditional cardiovascular events [59,105]. In parallel, SELECT showed semaglutide 2.4 mg reduces MACE by ~20% in patients with established CVD and overweight/obesity without diabetes, creating a new secondary-prevention lever whose optimal timing vis-à-vis an index AMI is still unknown [61]. Ongoing studies include *post-MI* GLP-1RA trials powered for MACE and pragmatic SGLT2-inhibitor strategies targeting HF-prone phenotypes.

### 8.7. Imaging, Surveillance, and Less-Stent Strategies

For complex PCI, evidence continues to accrue that intravascular imaging improves technical results and, in selected settings, outcomes: ILUMIEN IV (OCT-guided PCI) increased minimal stent area and lowered serious MACE at 2 years vs. angiography alone, while ECLIPSE suggests improved 1-year outcomes with IVUS guidance in severely calcified lesions. Whether these gains extend to ACS across the board beyond complex/calcified lesions remains open [38,106]. Elsewhere, the PULSE randomized trial found that routine 6-month CCTA after left-main PCI did not reduce an 18-month composite of death, spontaneous MI, unstable angina, or stent thrombosis vs. symptom-/ischemia-driven follow-up, though fewer spontaneous MIs were observed alongside more imaging-triggered revascularizations—an invitation to target higher-risk subgroups rather than embrace universal surveillance [39]. Finally, OCT-guided no-stent approaches for plaque erosion continue to mature (EROSION programme), reducing stent use without early safety signals, but definitive MACE-powered trials are still needed before broad adoption in ACS pathways [107].

### 8.8. Gender- and Phenotype-Specific Secondary Prevention

Two large contemporary RCTs in post-MI patients with preserved or mildly reduced LVEF diverged; REBOOT showed no benefit to chronic beta-blockade overall, whereas BETAMI–DANBLOCK suggested event reduction differences that may reflect populations, drug choice, and analytic nuances. Notably, prespecified and subsequent analyses highlight possible sex differences, with signals for harm among some women in REBOOT, emphasizing the need for sex-specific dosing and deprescription trials [58,108]. Ethnicity also matters as an updated East Asian antiplatelet consensus statement currently documents a higher bleeding/lower ischaemic risk trade-off (“East Asian paradox”) and supports a tailored antithrombotic intensity and duration for future trials [86]. Contemporary registry data (PRAISE) show that 1-year differences between STEMI and NSTEMI largely attenuate after adjustment for baseline risk and treatment patterns, emphasizing complete revascularization and secondary prevention for all ACS rather than using an electrocardiographic label as drivers of prognosis [109].

### 8.9. Surgical Secondary Prevention and Graft Biology

Beyond antiplatelets, NEWTON-CABG (CardioLink-5) showed that adding evolocumab to statins after CABG did not lower 2-year saphenous-vein graft (SVG) disease rates despite ~50% LDL-C reductions, implying that early SVG failure is less LDL-driven than thrombotic/inflammatory or mechanical failure, and that new targets (such as graft-bed inflammation, endothelial healing) merit exploration [54].

## 9. Simple Summary

This review synthesizes pivotal advancements in Acute Coronary Syndrome (ACS) management from 2023 to 2025, moving beyond the foundational 2023 ESC guidelines toward a more personalized, risk-stratified approach. Key developments include refined antithrombotic strategies, where new trials support early aspirin withdrawal after one month in select, low-risk MI patients, transitioning to potent P2Y_12_-inhibitor monotherapy to reduce bleeding without increasing ischaemic events. Revascularization strategy is also evolving, with recent evidence favouring culprit-only PCI in STEMI with multivessel disease, followed by staged in-hospital revascularization, especially in haemodynamically compromised patients. In the setting of cardiogenic shock, early implantation of Impella CP in patients with left ventricular failure due to STEMI may improve six-month as well as long-term mortality, however at the expense of higher complication rates including bleeding, ischaemia, and sepsis, advocating highly selective use. Furthermore, the 2025 ESC/EAS lipid guideline update advocates for immediate, in-hospital initiation of intensive combination therapy (high-intensity statin plus ezetimibe) to rapidly achieve aggressive LDL-C targets. These shifts are complemented by a growing emphasis on tailored secondary prevention, including the selective use of SGLT2 inhibitors, GLP-1 receptor agonists, and anti-inflammatory agents like colchicine based on individual patient profiles. By integrating these evidence-based, nuanced updates, this review provides a contemporary framework for clinicians to deliver precise, patient-centred care across the entire ACS spectrum.

## Figures and Tables

**Table 1:** Summary of recent clinical trials contributing to guideline update.

Year of Publication	Trial	Conclusion	Guideline Contribution
2020	TICO (ACS PCI)	Ticagrelor monotherapy after 3 months reduced NACE vs. 12 months DAPT.	Supports shorter DAPT with early ticagrelor monotherapy in selected ACSs.
2021	FLOWER-MI	No superiority of FFR-guided strategy for 1-year MACE.	Either approach acceptable; no mandate to use FFR for non-culprit PCI in STEMI.
2021	MASTER-DAPT (HBR, ~50% ACS)	Noninferior NACE/MACCE; less major/CRNM bleeding with 1-month DAPT.	Underpins shortened DAPT in high bleeding risk (including ACS subsets).
2021	ASSAIL-MI	Increased myocardial salvage; no definitive infarct-size/MACE reduction.	Mechanistic support for IL-6 axis targeting; not practice-changing alone.
2021	IAMI	Reduced composite of death/MI/stent thrombosis at 12 months.	Supports in-hospital influenza vaccination post-MI.
2022	PACMAN-AMI	Greater plaque regression/stabilization in non-culprit vessels.	Backs early, intensive LDL-C lowering; informs 2025 dyslipidaemia intensification.
2022	SECURE	Reduced MACE and cardiovascular death.	Supports implementation tools (polypill) for secondary prevention.
2023	ILUMIEN IV	Larger minimum stent area; no overall TVF difference at 2 years.	Encourages intravascular imaging for optimization, especially in complex lesions.
2023	MULTISTARS AMI	Immediate strategy superior to weeks-later staging in stable STEMI.	Supports completion during index setting over late staging (with careful selection).
2023	CLEAR Outcomes	Reduced MACE; robust LDL-C and hs-CRP reductions.	Adds bempedoic acid to options for statin-intolerant ACS survivors.
2023	ECLS-SHOCK (AMI shock)	No 30-day mortality reduction; more complications.	No routine VA-ECMO in AMI shock; reserve for rescue/selected cases.
2024	EMPACT-MI	Neutral primary (death/first HF hospitalization); fewer HF hospitalizations; good safety.	Permits targeted SGLT2i use (HF-prone phenotypes) rather than universal early use.
2024	DAPA-MI (no diabetes/HF)	Win-ratio benefit for cardiometabolic outcomes; no MACE reduction at ~1 year.	Signals metabolic benefits; MACE-powered post-MI trials still needed.
2025	REBOOT-CNIC	No overall benefit; possible benefit in LVEF at 40–49%; potential sex differences.	Suggests no routine long-term beta-blocker at LVEF ≥ 50%; individualize at 40–49%.
2025	BETAMI–DANBLOCK	Lower death/MACE (HR ≈ 0.85); effect stronger at LVEF 40–49%.	With REBOOT, supports LVEF-stratified use (benefit mainly at 40–49%).
2025	OPTION-STEMI	Noninferiority not shown for immediate strategy; concerns in patients with HF signs.	Favours culprit-only acutely with index-stay staging in many STEMI patients.
2025	TARGET-FIRST (low-risk MI, fully revascularized)	Noninferior ischaemic outcomes; less bleeding with aspirin stopping at 1 month.	Enables 1-month aspirin discontinuation in carefully selected MI patients.
2025	NEO-MINDSET (ACS PCI)	Noninferiority not met; early ischaemic hazard despite lower bleeding.	Do not drop aspirin immediately after ACS-PCI in unselected patients.
2025	DUAL-ACS (all-comer MI)	Similar ischemia; less bleeding at 15 months; pragmatic; not powered for noninferiority.	Supports 3-month DAPT when bleeding risk predominates (hypothesis-generating).
2025	HELP-MI SWEDEHEART	No significant reduction in upper GI bleeding at 1 year overall.	Keep PPI co-therapy first-line; consider targeted H. pylori testing only.
2025	TACSI (post-CABG for ACS)	No ischaemic benefit; increased major bleeding with DAPT.	Endorses aspirin alone routinely after CABG for ACS.
2025	TOP-CABG	Noninferior SVG occlusion; less clinically relevant bleeding.	Suggests time-limited intensification then aspirin alone post-CABG.
2025	NEWTON-CABG (CardioLink-5)	No reduction in 2-year SVG disease despite ~50% LDL-C drop.	Routine PCSK9 solely for SVG patency not justified; LDL-lowering remains for event risk.
2025	DAPT-SHOCK AMI	Immediate platelet inhibition; noninferiority not met for 30-day composite; favourable secondary signals.	Reasonable bridge when enteral absorption is unreliable; outcome benefit unproven.
2025	PULSE (LM-PCI follow-up)	Negative primary; fewer spontaneous MIs with more imaging-triggered revascularization; no TLR difference.	No routine CCTA after LM-PCI; selective imaging only.

## Data Availability

The original contributions presented in this study are included in the article. Further inquiries can be directed to the corresponding author.
